# Anti-obesity effects of chikusetsusaponins isolated from *Panax japonicus *rhizomes

**DOI:** 10.1186/1472-6882-5-9

**Published:** 2005-04-06

**Authors:** Li-Kun Han, Yi-Nan Zheng, Masayuki Yoshikawa, Hiromichi Okuda, Yoshiyuki Kimura

**Affiliations:** 1 Faculty of Environmental and Symbiotic Sciences, Prefectural University of Kumamoto, Tsukide, Kumamoto 862-8502, Japan; 2 Department of Chinese Material Medicine, Chinese Material Medicine College of Jilin Agricultural University, Changchun-City, Jilin 130118, China; 3 Department of Pharmacognosy, Kyoto Pharmaceutical University, Yamashina, Kyoto 607-8412, Japan; 4 Division of Biochemistry, Department of Molecular and Cellular Biology, School of Medicine, Ehime University, Shitsukawa, Toon-City, Ehime 791-0295, Japan

## Abstract

**Background:**

The rhizomes of *Panax japonicus *are used as a folk medicine for treatment of life-style related diseases such as arteriosclerosis, hyperlipidemia, hypertension and non-insulin-dependent diabetes mellitus as a substitute for ginseng roots in China and Japan. Obesity is closely associated with life-style-related diseases. This study was performed to clarify whether chikusetsusaponins prevent obesity induced in mice by a high-fat diet for 9 weeks.

**Methods:**

We performed two *in vivo *experiments. In one, female ICR mice were fed a high-fat diet with or without 1 or 3% chikusetsusaponins isolated from *P. japonicus *rhizomes for 9 weeks. In the other, lipid emulsion with or without chikusetsusaponins was administered orally to male Wistar rats, and then the plasma triacylglycerol level was measured 0.5 to 5 h after the orally administered lipid emulsion. For *in vitro *experiments, the inhibitory effects of total chikusetsusaponins and various purified chikusetsusaponins on pancreatic lipase activity were determined by measuring the rate of release of oleic acid from triolein in an assay system using triolein emulsified with lecithin.

**Results:**

Total chikusetsusaponins prevented the increases in body weight and parametrial adipose tissue weight induced by a high-fat diet. Furthermore, consumption of a high-fat diet containing 1 or 3% total chikusetsusaponins significantly increased the fecal content and triacylglycerol level at day 3 compared with the high-fat diet groups. Total chikusetsusaponins inhibited the elevation of the plasma triacylglycerol level 2 h after the oral administration of the lipid emulsion. Total chikusetsusaponins, chikusetsusaponin III, 28-deglucosyl-chikusetsusaponin IV and 28-deglucosyl-chikusetsusaponin V inhibited the pancreatic lipase activity.

**Conclusion:**

The anti-obesity effects of chikusetsusaponins isolated from *P. japonicus *rhizomes in mice fed a high-fat diet may be partly mediated through delaying the intestinal absorption of dietary fat by inhibiting pancreatic lipase activity. The present study clearly indicated that the saponin fractions of *P. japonicus *rhizomes had a significant anti-obesity action and supports the traditional usage as a substitute drug for ginseng roots.

## Background

The rhizomes of *Panax japonicus *C.A. Meyer (Japanese name; Chikusetsuninjin), have been used as a substitute for Ginseng roots (the roots of *Panax ginseng *C.A. Meyer). On the other hand, Ginseng roots are used as the remedy for life-style-related diseases such as arteriosclerosis hyperlipidemia, hypertension and non-insulin-dependent diabetes mellitus in China, Korea, Japan and Europe and there are a number of reports on the pharmacological studies of Ginseng roots [1-8]. It has been reported that the rhizomes of *Panax japonicus *have anti-ulcer action and fibrinolysis [9,10]. However, the investigations of pharmacological effects of *Panax japonicus *rhizomes on life-style-related diseases such as obesity, arteriosclerosis hyperlipidemia, hypertension and non-insulin-dependent diabetes mellitus have been not thoroughly reported. Obesity is one of the fastest-growing major diseases in many areas of the world including Europe, the United States and Japan. Obesity results from an imbalance between energy intake and expenditure. Obesity is closely associated with life-style-related diseases such as hyperlipidemia, hypertension, arteriosclerosis and non-insulin-dependent diabetes mellitus and with increased risk of coronary heart disease [11]. It has been reported that variations in total energy intake and diet composition are important in the regulation of metabolic processes [12,13]. Furthermore, it has been suggested that dietary fat promotes body fat storage more effectively than dietary carbohydrate. Thus, inhibition of the digestion and absorption of dietary fat is a key to treating obesity. Dietary fat is not directly absorbed from the small intestine unless it has been subjected to the action of pancreatic lipase [14]. In this study, we examined the effects of total chikusetsusaponins of *P. japonicus *rhizomes on obesity induced by long-term feeding of a high-fat diet.

## Methods

### Materials

Pancreatic lipase was purchased from Sigma Chemical Co. (St Louis, MO). Triglyceride E- and Total Cholesterol E-test kits were purchased from Wako Pure Chemical Co. (Osaka, Japan). Laboratory pellet chow was purchased from CLEA Japan (Osaka, Japan). Beef tallow, casein, vitamin and mineral mixtures were purchased from Oriental Yeast Co. Ltd (Tokyo, Japan). Orlistat (a lipase inhibitor) was purchased from Hong Kong Market. Other chemicals were of reagent grade.

### Plant materials

The rhizomes of *P. japonicus *were obtained from Tochimoto Tennkaido Co. Ltd. (Osaka, Japan). Voucher specimens (No. PJ020817) are deposited in the Faculty of Environmental and Symbiotic Sciences, Prefectural University of Kumamoto and the Second Department of Medical Biochemistry, School of Medicine, Ehime University.

### Preparation of total saponins and five chikusetsusaponins from the rhizomes of *P. japonicus*

**Figure 1:**
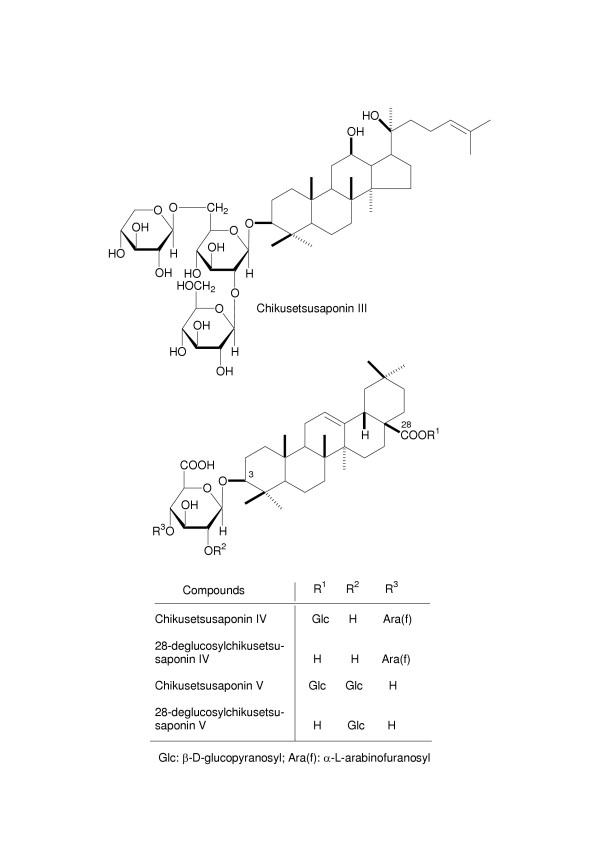
The structure of various chikusetsusaponins

Chikusetsusaponins were isolated using by a modification of previous reported method [15,16]. The dried rhizomes of *P. japonicus *(1 kg) were extracted three times by refluxing with methanol (3 L). After removal of the solvent from the methanol solution under reduced pressure, the extract (370 g) was subjected to chromatography on reversed-phase highly porous polymer [DIAION HP-20 (4 kg), Mistubishi Chemical Ind. Ltd.: elution with H_2_O (800 mL), MeOH (500 mL), and CHCl_3 _(300 mL)] to provide the three fractions such as H_2_O eluate (235 g), MeOH eluate (130 g), and CHCl_3 _eluate (5 g). The fraction eluted with methanol was designated the "total chikusetsusaponins". The MeOH eluate (total saponin fraction, 110 g) was separated by column chromatography on silica gel [silica gel G (2 kg), Merck, CHCl_3_-MeOH-H_2_O] to furnish four fractions and the fractions 2 and 3 were purified by HPLC (YMC-pack ODS, MeOH-H_2_O-AcOH) to give chikusetsusaponins III (13.5 g), IV (6.2 g) and V (54.5 g), 28-deglucosylchikusetsusaponins IV (15 mg) and V (110 mg). 28-Deglucosylchikusetsusaponins IV and V were also obtained by alkaline hydrolysis of chikusetsusaponins IV and V, respectively. The isolated five compounds were identified by comparison of their physical data ([α]_D_, IR, ^1^H- and ^13^C-NMR) with reported values [15,16]. The purity of each compound was over 95% based on the analysis by HPLC (Fig. 1).

### Diet compositions

Previously, we reported that varying the casein concentration (22, 31, 34 and 36 % diets) in high-fat diet containing 40 % beef tallow did not affect body weight or parametrial adipose tissue weight [17]. Therefore, we substituted 1 or 3% total chikusetsusaponins isolated from *P. japonicus *rhizomes for an equivalent mass of casein in the high-fat diets shown in Table 1. To avoid autooxidation of the fat components, food was stored at -30°C. If the diet except fat from high-fat diet was used as control diet, carbohydrate (*e.g*. corn starch or sugar) instead of fat was added to the diet; and consequently this diet is high-carbohydrate diet. Black et al. reported that feeding high-carbohydrate diet containing 73% kcal corn starch for 16 weeks elevated the α-disaccharidase activity in small intestine [18]. Therefore, it seems likely that the high-carbohydrate diet may affect carbohydrate metabolism. In this study, we used laboratory chow diet as a control diet.

**Table 1:** Composition of experimental high-fat diets

	Total chikusetsusaponins (g/100 g diet)				
	
	High-fat diet (HF)	HF + 1% chikusetusaponins	HF + 3% chikusetsusaponins	HF + 0.012% orlistat	HF + 0.024% orlistat
Beef tallow	40	40	40	40	40
Corn starch	10	10	10	10	10
Sugar	9	9	9	9	9
Mineral mixture	4	4	4	4	4
Vitamin mixture	1	1	1	1	1
Casein	36	35	33	36	36
Total saponins	0	1	3	0	0
Orlistat	0	0	0	0.012	0.024

### Animals

Female ICR strain mice (3 weeks old) and male Wistar King strain rats (6 weeks old) were obtained from CLEA Japan (Osaka, Japan) and Charles River Japan (Yokohama, Japan), respectively, and housed for 1 week under a 12 h/12 h light/dark cycle in a temperature- and humidity-controlled room.

The animals were given free access to food and water. After adaptation to the lighting conditions for 1 week, the healthy animals were used in these experiments. The experimental protocol was approved by the Animal Studies Committees of Ehime University and Kumamoto Prefectural University.

### Estimation of body and parametrial adipose tissue weights, plasma triacylglycerol and total cholesterol in mice fed a high-fat diet for 9 weeks

Female ICR mice (3 weeks old) were divided into four groups that were matched for body weight, after 1 week of being fed laboratory pellet chow *ad libitum*. The control group continued to be fed laboratory pellet chow *ad libitum*. The mice consumed the high-fat diet or the high-fat diet containing 1 and 3% total chikusetsusaponins or 0.012 and 0.024% orlistat (positive control) for 9 weeks. The body weight of each mouse was measured once a week and the total amount food consumed was recorded 3 times per week. After the mice had been fed these diets for 9 weeks, blood was taken from each mouse by venous puncture under anesthesia with diethyl ether; the mice were then killed with an overdose of diethyl ether. Experiments were performed in a ventilated room. The plasma was prepared and frozen at -80°C until analysis. The liver and parametrial adipose tissue were dissected and weighed. Liver triacylglycerol and total cholesterol concentrations were measured using Wako Triglyceride E-Test and Total Cholesterol E-Test kits.

### Fat excretion in feces of mice

Female ICR mice (3 weeks old) were housed for 1 week in a room maintained at 25 ± 1°C with 60% relative humidity and given free access to standard laboratory pellet chow and water. The mice consumed the high-fat diet or the high-fat diet containing 1% or 3% total chikusetsusaponins, or 0.025% orlistat for 3 days. Wet weight and triacylglycerol content in the feces obtained during the last 24 h were measured using the Wako Triglyceride E-Test kit.

### Plasma triacylglycerol levels after oral administration of lipid emulsions to rats

Male Wistar rats were also housed for 1 week in the above same conditions. After rats had been deprived of food overnight, they were orally administered 1 mL of a lipid emulsion consisting of corn oil (3 mL), cholic acid (40 mg) and cholesteryl oleate (1 g) plus physiological saline (3 mL), the lipid emulsion (1 mL) plus total chikusetsusaponins (final concentration 1000 mg/kg body) or the lipid emulsion plus orlistat (final concentration 45 mg/kg body). Blood samples were taken from the tail vein 0, 0.5, 1, 2, 3, 4 and 5 h after administration of the lipid emulsion with or without total saponins or orlistat using a capillary tube (heparinized), and centrifuged at 5500 × g for 5 min in a Model KH-120 M (Kubota Co., Osaka, Japan) centrifuge to obtain the plasma. The plasma triacylglycerol concentration was determined using a Wako Triglyceride E-Test kit.

### *In vitro *pancreatic lipase activity

Lipase activity in the porcine pancreas was assay as described previously [19]. Enzyme activity was expressed as μmoles of oleic acid released per mL of reaction mixture per min.

### Statistical analysis

All values are expressed as means ± s.e. Data were analyzed by one-way ANOVA, and then differences among means were analyzed using the Fisher's protected LSD test. Differences were considered significant at *P *< 0.05.

## Results

### Fat excretion in feces of mice fed a high-fat diet with or without total chikusetsusaponins

Consumption of the high fat diet for 3 days reduced the feces weight (0.14 ± 0.035 g/mouse/day) at day 3 compared with that of the control group (1.04 ± 0.017 g /mouse/day). Mice fed the high-fat diet plus 3% total chikusetsusaponins, or 0.025% orlistat for 3 days had significantly higher triacylglycerol level at day 3 compared with the high-fat diet group (Table 2).

**Table 2:** Effects of total chikusetsusaponins and orlistat (a lipase inhibitor) on fat excretion on day 3 in feces of mice fed a high-fat diet

	Animal No.	Feces weight (g)	Triacyglycrol in feces (μmol/g feces)
Laboratory chow pellet	13	1.04 ± 0.017*	5.05 ± 0.70*
High-fat diet (HF)	13	0.14 ± 0.035	36.66 ± 1.03
HF +			
1% Total chikusetsusaponins	7	0.26 ± 0.012*	41.64 ± 3.48
HF +			
3% Total chikusetsusaponins	7	0.31 ± 0.049	53.42 ± 4.86*
HF +			
0.025 % orlistat (lipase inhibitor)	7	0.22 ± 0.040	394.38 ± 65.39*

Values are means ± S.E. of 7 – 13 mice. * Significantly different from the high-fat diet group, *P *< 0.05.

### Food consumption; body, parametrial adipose tissue and liver weights; and triacylglycerol content in the livers of mice fed high-fat diet with or without chikusetsusaponins for 9 weeks

**Figure 2:**
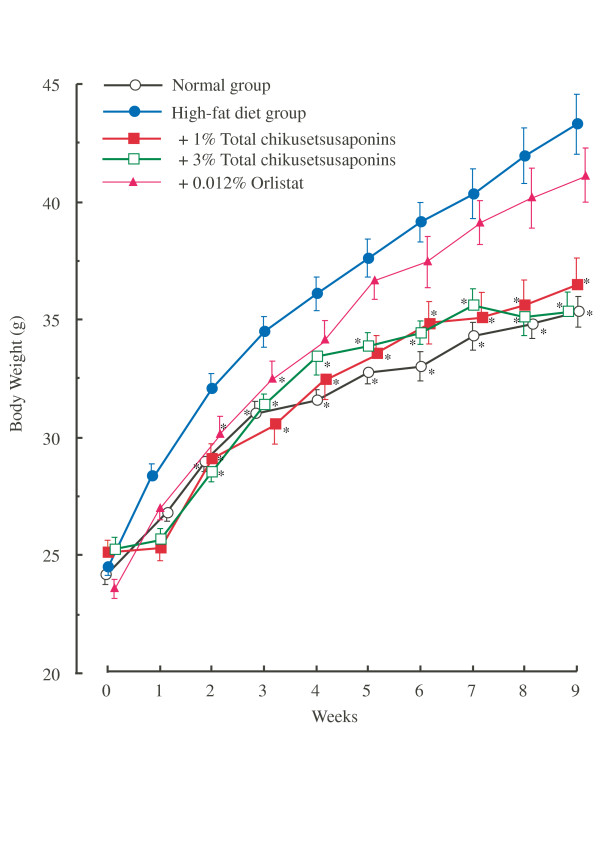
**Effects of chikusetsusaponins and orlistat (a lipase inhibitor) on body weight in mice fed a high-fat diet for 9 weeks**. Open circles, normal groups; solid circles, high-fat diet groups; open squares, high-fat plus 1% total chikusetsusaponin diet groups; solid squares, high-fat plus 3% total chikusetsusaponin diet groups; solid triangle, high-fat plus 0.012% orlistat diet. Values are means ± S.E. of 11–26 mice. * Significantly different from the high-fat diet group, *P *< 0.05.

The mean food consumption per week per mouse was significantly different between the control group and high-fat diet groups, being 409.4 ±7.0 KJ in the control group and 611.0 ± 41.6 KJ in the high-fat diet group?? but was not significantly different among the high-fat diet groups, high-fat plus 1% and 3% saponin-diet groups, and high-fat plus 0.012% orlistat-diet groups, being 611.0 ± 41.6 KJ (high-fat diet), 708.7 ± 34.6 KJ (high-fat diet plus 1% chikusetsusaponin), 686.0 ± 30.0 KJ (high-fat diet plus 3% chikusetsusaponin) and 693.1 ± 53.8 KJ (high-fat diet plus 0.012% orlistat). A high-fat plus 0.024% orlistat diet significantly reduced the body weight to 4 weeks compared to both the normal and the high-fat diet groups, but among 15 mice of high-fat plus 0.024% orlistat-diet groups, 10 mice died by the severe diarrhea until 5 weeks by the feeding high-fat diet containing 0.024% orlistat (data not shown). Orlistat is a potent inhibitor of pancreatic lipase; therefore, the absorption of dietary fat from small intestine was strongly inhibited by feeding high-fat diet containing 0.025% orlistat (Table 2). Mice treated with high-fat diet plus 0.024% orlistat for a long term died by severe diarrhea, but high-fat diet plus 0.012% orlistat-fed mice did not cause diarrhea (data not shown). This finding suggests that feeding level of 0.024% orlistat in the high-fat diet to mice for a long term may cause the physiological toxicity through the continuous inhibition of pancreatic lipase. Feeding high-fat plus 0.012% orlistat diet had no effect on the adipose tissue weight. In the present study, the discrepancy between anti-obesity action and inhibitory action of pancreatic lipase by orlistat could not sufficiently be clarified; therefore, this discrepancy is needed to clarify in future studies. Figure 2 shows the changes in body weight of the groups during the experiment. Consuming a high-fat diet for 9 weeks caused significant increases in body weight at 1 to 9 weeks compared to that of the control group (laboratory pellet chow). Consuming a high-fat diet supplemented with 1% or 3% chikusetsusaponins significantly suppressed the increase in body weight during the experimental period compared to the high-fat diet, but the high-fat diet plus 0.012% orlistat did not affect. The final parametrial adipose tissue weights of the groups are shown in Table 3. The final parametrial adipose tissue weight was significantly increased by consumption of a high-fat diet compared to the control diet, and that in animals with a high-fat diet containing 1% or 3% chikusetsusaponins was significantly reduced as compared to that in the high-fat diet group. Furthermore, mice fed a high-fat diet long term developed fatty liver, with increases of the liver weight and an accumulation of hepatic triacylyglycerol compared to the control group. Consumption of a high-fat diet containing 1% or 3% chikusetsusaponins, or 0.012% orlistat reduced the liver weight and hepatic triacylglycerol content compared to consumption of a high-fat diet group (Table 3).

**Table 3:** Effects of total chikusetsusaponins and orlistat (a lipase inhibitor) on liver weight, hepatic triacylglycerol, and adipose tissue weight in mice fed a high-fat diet for 9 weeks.

	Animal No.	Liver weight (g/100 g body weight)	Hepatic triacylglycerol (μmol/g)	Parametrial adipose tissue weight (g)
Laboratory chow pellet	26	5.20 ± 0.46*	22.3 ± 2.3*	0.71 ± 0.1*
High-fat diet (HF)	26	6.35 ± 0.76	141.8 ± 21.8	1.59 ± 0.3
HF +				
1% Total saponins	11	6.13 ± 0.79	101.5 ± 6.8*	0.87 ± 0.1*
HF +				
3% Total saponin	11	5.79 ± 0.47*	83.3 ± 6.7*	0.74 ± 0.1*
HF + 0.012% Orlistat	15	4.00 ± 0.11*	101.1 ± 4.8*	2.03 ± 0.12

Values are means ± S.E. of 11 – 26 mice. *Significantly different from the high-fat diet group, *P *< 0.05.

### Total chikusetsusaponins reduced the elevation of rat plasma triacylglycerol levels after oral administration of lipid emulsion to rats

**Figure 3:**
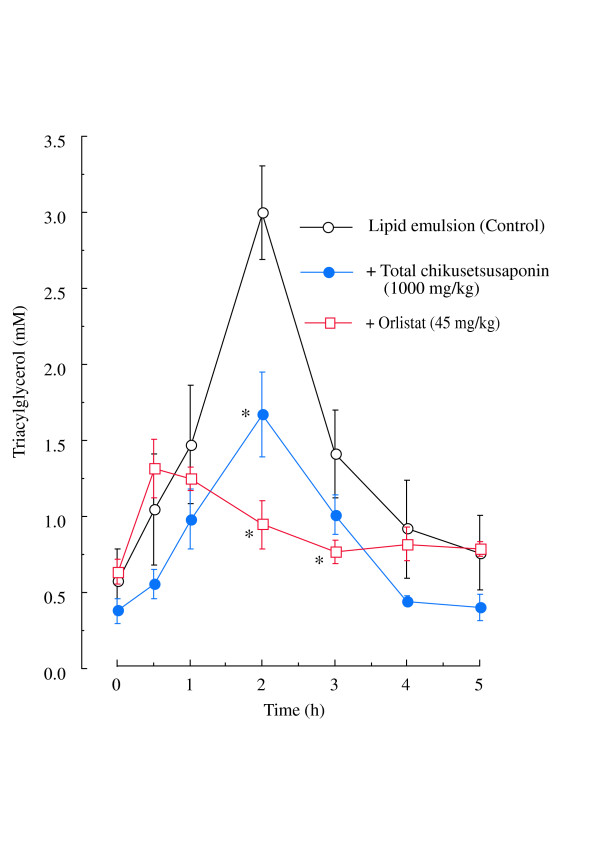
**Effects of total chikusetsusaponins and orlistat (a lipase inhibitor) on rat plasma triacylglycerol levels after oral administration of a lipid emulsion**. Open circles, lipid emulsion; solid circles, lipid emulsion plus total chikusetsusaponins (1000 mg/kg) and open squares, lipid emulsion plus orlistat (a lipase inhibitor) (45 mg/kg). Values are means ± S.E. of 4 rats. * Significantly different from lipid-emulsion-only treated group, *P *< 0.05.

The plasma triacylglycerol level (2.98 ± 0.31 mM, n = 4) at 2 h after orally administered lipid-emulsion was significantly reduced to 1.66 ± 0.28 mM (n = 4) and 0.93 ± 0.16 mM (n = 4), respectively, by the oral administration of total chikusetsusaponins (1000 mg/kg) and orlistat (45 mg/kg) (Fig. 3).

### Effects of total chikusetsusaponins and various purified chikusetsusaponins on pancreatic lipase activity *in vitro*

**Figure 4:**
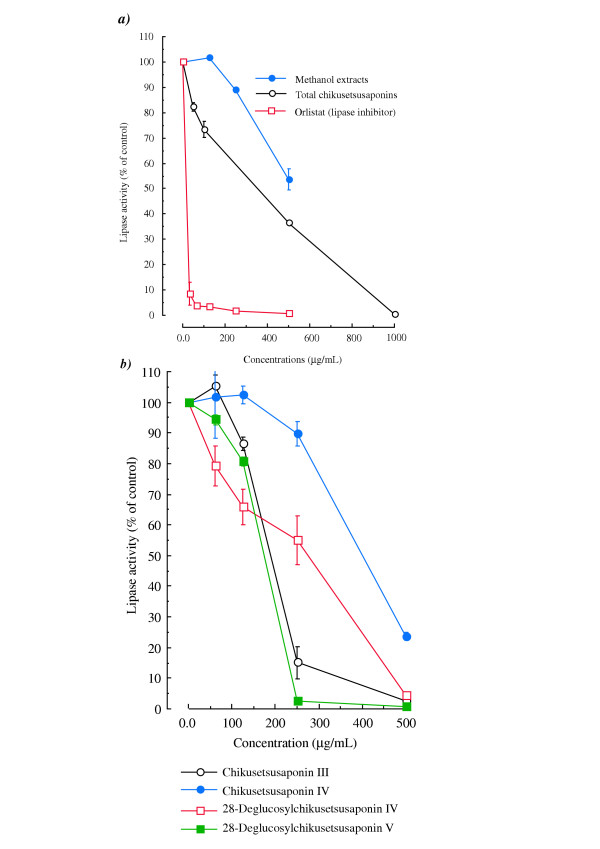
**Effects of total chikusetsusaponins, various chikusetsusaponins and orlistat (a lipase inhibitor) on pancreatic lipase activity**. Values are means ± S.E. of 4 experiments. a) Solid circles, methanol extract; open circles, total chikusetsusaponins; open squares, orlistat (a lipase inhibitor) was used as a positive control. b) Open circles, chikusetsusaponin III; solid circles, chikusetsusaponin IV; open squares, 28-deglucosyl-chikusetsusaponin IV; solid squares, 28-deglucosyl-chikusetsusaponin V.

As shown in Fig. 4a, methanol extracts, total chikusetsusaponins and the positive control orlistat dose dependently inhibited pancreatic lipase activity in an assay system using triolein emulsified with lecithin, but water and CHClS_3_-eluate fraction had no effect. We then examined the various chikusetsusaponins isolated from *P. japonicus *rhizomes. Chikusetsusaponin III and 28-deglucosylchikusetsusaponins IV and V dose-dependently inhibited the pancreatic lipase activity at concentrations of 125 to 500 μg/mL (Fig. 4b).

## Discussion

There are a number of reports showing that ginseng roots are clinically anti-allergic, anti-hypertensive, hypoglycemic, anti-thrombotic, and anti-arteriosclerotic [7,8]. Recently, obesity is increasing in advanced countries including Europe, the United States and Japan. Obesity is closely associated with life-style-related diseases such as hyperlipidemia, hypertension, arteriosclerosis and non-insulin-dependent diabetes mellitus and with increased risk of coronary heart disease [11]. Although the rhizomes of *P. japonicus *are used as a substitute drug for ginseng roots in Japan, the pharmacological effects of these rhizomes on life-style-related diseases have been not clarified. A methanol extract of these rhizomes and chikusetsusaponins such as chikusetsusaponins III, IV and V derived from *P. japonicus *rhizomes have been shown to promote the fibrinolysis [10]. Yamahara et al. [9] reported that the saponin fractions and chikusetsusaponin III of *P. japonicus *exert anti-ulcer effects. However, there have hitherto been no reports on the inhibitory effects of chikusetsusaponins isolated from *P. japonicus *rhizomes on obesity induced by consumption of a high-fat diet. Dietary fat can increase body weight and adiposity in humans and animals more effectively than dietary carbohydrate [17,20-22]. Dietary fat is not directly absorbed from the intestine unless it has been subjected to the action of pancreatic lipase [14]. Therefore, the application of pancreatic lipase inhibitor was examined earlier as a treatment for high-fat diet-induced obesity in humans. It has been reported that a pancreatic lipase inhibitor, orlistat prevented obesity and hyperlipidemia through the enhancement of fat excretion in feces and the inhibition of pancreatic lipase [23]. We found that feeding high-fat diet containing 40% fat content caused obesity [17,19], but the high-fat diet containing 25% fat content slightly increased the body weight (data not shown). Anai et al. reported that feeding high-fat diet containing 60% fat for 2 weeks slightly increased the body weight [24]. Furthermore, it has been reported that feeding high-fat diet containing 58% kcal fat for 16 weeks caused obesity [18]. In the present study, to examine the effects of chikusetsusaponins on high-fat diet-induced obesity, we used the obesity model induced by feeding high-fat diet containing 40% fat for 9 weeks. We found that the administration of total chikusetsusaponins isolated from *P. japonicus *significantly suppressed the increase in body weight in mice fed a high-fat diet containing 40% beef tallow for 9 weeks. The treatment with total chikusetsusaponins also significantly reduced the final parametrial adipose tissue weight compared to that of the high-fat diet group. These inhibitions did not depend on decreased food or energy intake, because there was no significant difference between these in the groups fed the high-fat diet and the high-fat diet containing 1 or 3 % total chikusetsusaponin. Next, we examined the effects of total chikusetsusaponins on plasma triacylglycerol concentrations after oral administration of a lipid emulsion in rats, and found that the total chikusetsusaponins reduced the elevation of plasma triacylglycerol levels as measured by an oral lipid emulsion tolerance test. This finding suggests that total chikusetsusaponins may inhibit the uptake of dietary fat. In fact, total chikusetsusaponins strongly inhibited the pancreatic lipase activity, and therefore, we attempted to isolate substance(s) from total saponins that inhibit pancreatic lipase activity. Five chikusetsusaponins were isolated from total saponin fractions and identified as chikusetsusaponins III, IV and V and 28-deglucosyl-chikusetsusaponins IV and IV by the direct comparison with authentic samples. Among these five saponins, chikusetsusaponin III and 28-deglucosylchikusetsusaponins IV and V inhibited the pancreatic lipase activity, most strongly. The contents of total chikusetsusaponins in the MeOH extract are about 35%, and the contents of chikusetsusaponins III, IV and V in the total saponin fraction are about 10.4, 4.8 and 41.9%, respectively. On the other hand, the content of 28-deglucosylchikusetsusaponins IV and V in the total saponins are about 0.01 and 0.08%, respectively. Chikusetsusaponins IV and V are rapidly converted to 28-deglycosyl-forms by the treatment with alkaline solution. Therefore, it seems likely that the above 28-deglucosylchikusetsusaponin derive from chikusetsusaponins IV and V through the small intestine after orally administered chikusetsusaponins IV and V. Consequently, it is suggested that their metabolites (28-deglucosyl form) exhibit the inhibition of pancreatic lipase activity.

## Conclusion

Total chikusetsusaponins isolated from *P. japonicus *may prevent high-fat-diet-induced increases in body weight and fat storage in adipose tissue by inhibiting intestinal absorption of dietary fat through the inhibition of pancreatic lipase activity, and the active components were identified here as chikusetsusaponins III and IV, 28-deglucosyl-chikusetsusaponins IV and V. The present study clearly indicated that the saponin fractions of *P. japonicus *rhizomes had a significant anti-obesity action and supports the traditional usage as a substitute drug for ginseng roots. Hence it might help in preventing obesity complications and serve as good adjuvant in the present armamentarium of anti-obesity drugs.

## Competing interests

The author(s) declare that they have no competing interests.

## Authors' contributions

The experimental design was made by L-K. Han, H. Okuda and Y. Kimura

The experiments of animal models and enzyme activity were worked by L-K. Han, H. Okuda and Y. Kimura. The isolation and structural determination of various chikusetsusaponins from *Panax japonicus *rhizomes were worked by L-K. Han, Y-N. Zheng and M. Yoshikawa.

The paper was written by L-K. Han and Y. Kimura.

## Pre-publication history

The pre-publication history for this paper can be accessed here:


